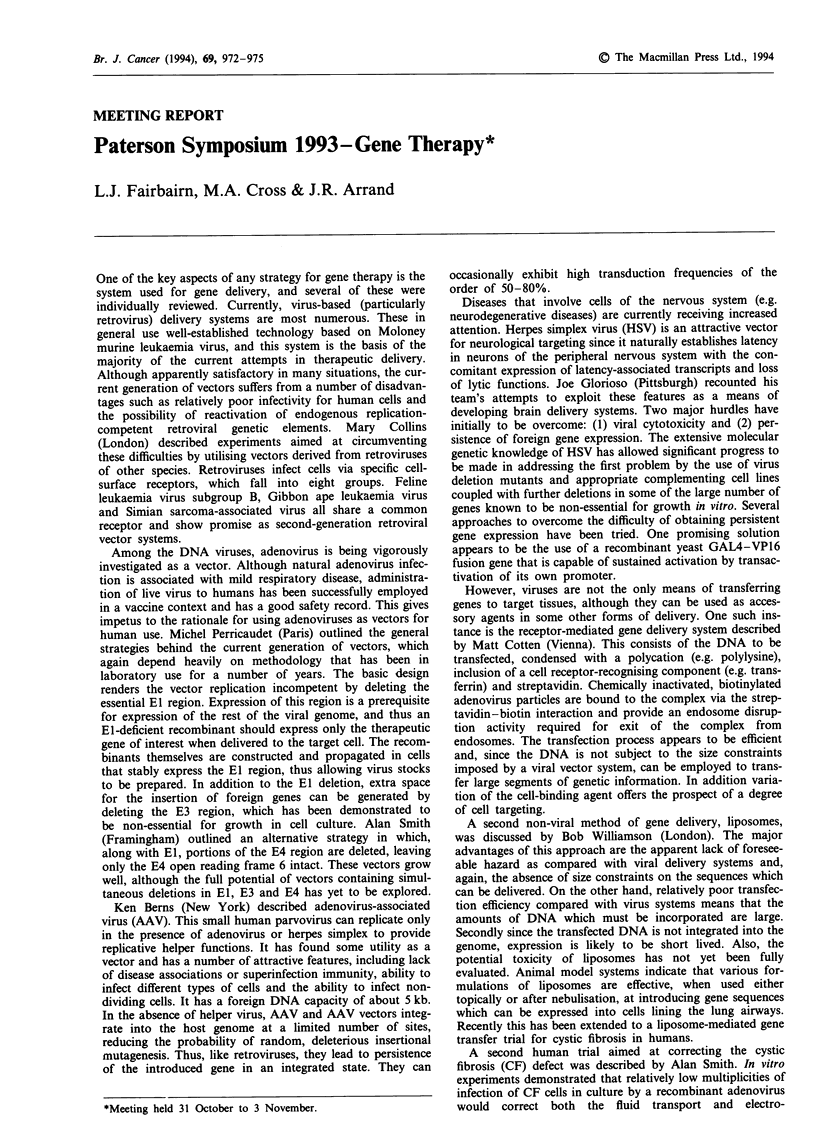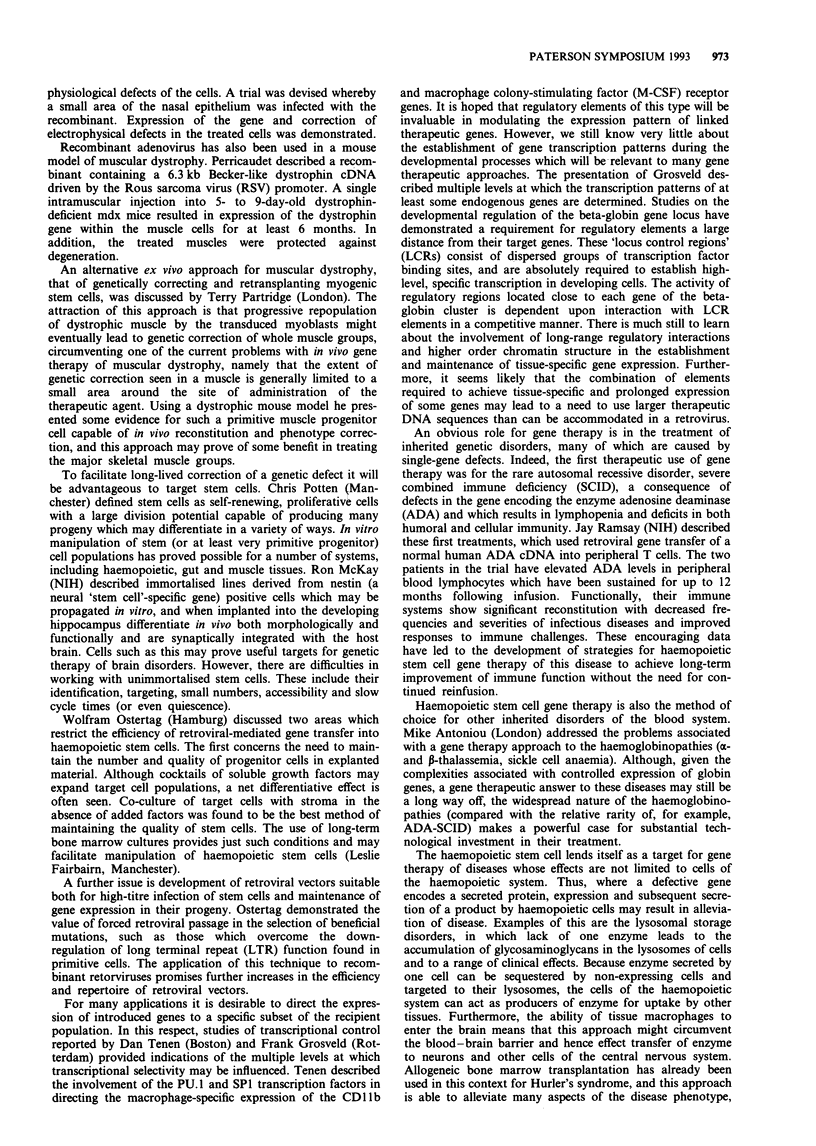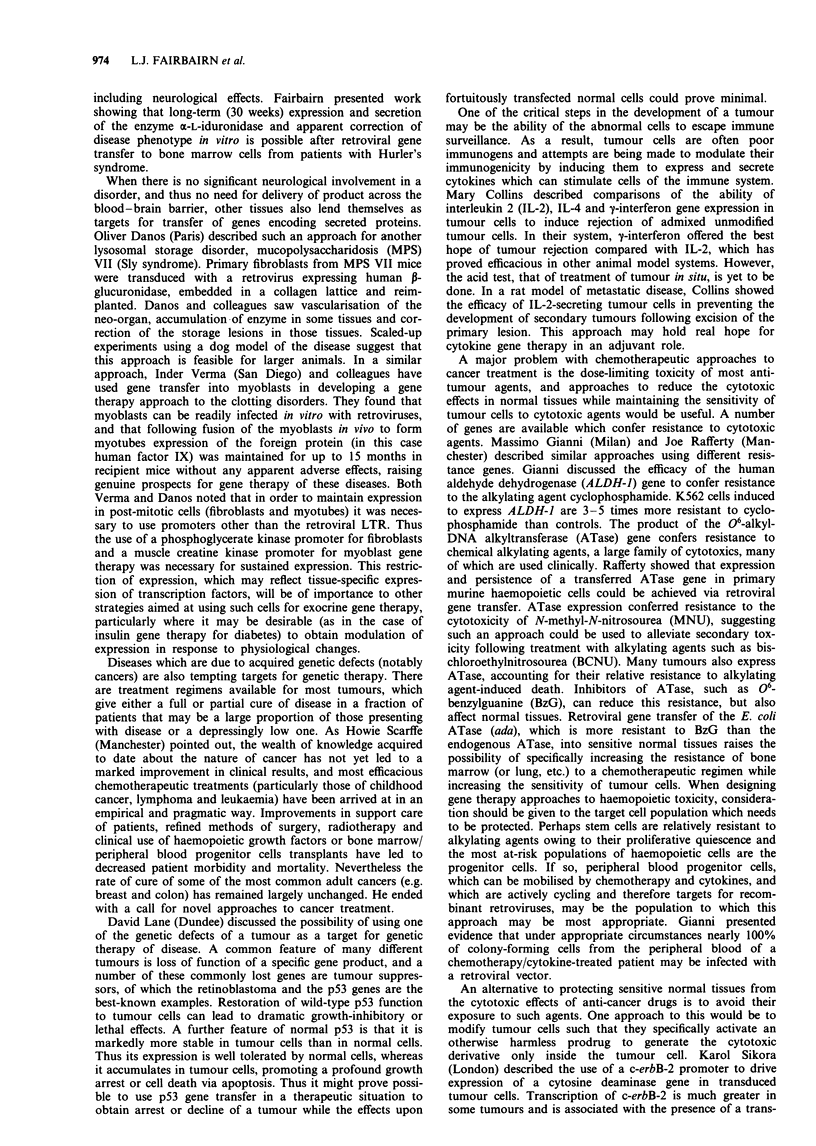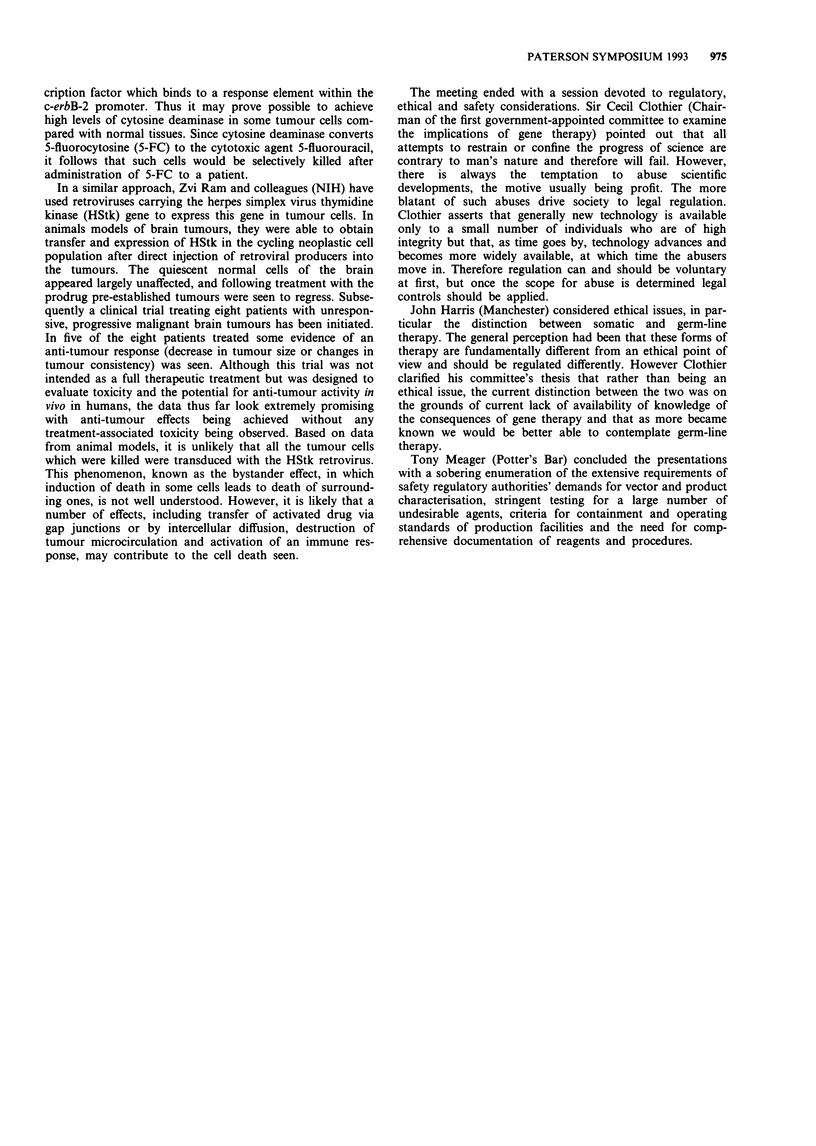# Paterson symposium 1993--gene therapy.

**DOI:** 10.1038/bjc.1994.188

**Published:** 1994-05

**Authors:** L. J. Fairbairn, M. A. Cross, J. R. Arrand


Br. J. Cancer (1994), 69, 972-975 © The Macmillan Press Ltd., 1994

MEETING REPORT

### Paterson Symposium 1993 – Gene Therapy*

L.J. Fairbairn, M.A. Cross & J.R. Arrand

One of the key aspects of any strategy for gene therapy is the system used for gene delivery, and several of these were individually reviewed. Currently, virus-based (particularly retrovirus) delivery systems are most numerous. These in general use well-established technology based on Moloney murine leukaemia virus, and this system is the basis of the majority of the current attempts in therapeutic delivery. Although apparently satisfactory in many situations, the current generation of vectors suffers from a number of disadvantages such as relatively poor infectivity for human cells and the possibility of reactivation of endogenous replication-competent retroviral genetic elements. Mary Collins (London) described experiments aimed at circumventing these difficulties by utilising vectors derived from retroviruses of other species. Retroviruses infect cells via specific cell-surface receptors, which fall into eight groups. Feline leukaemia virus subgroup B, Gibbon ape leukaemia virus and Simian sarcoma-associated virus all share a common receptor and show promise as second-generation retroviral vector systems.

Among the DNA viruses, adenovirus is being vigorously investigated as a vector. Although natural adenovirus infection is associated with mild respiratory disease, administration of live virus to humans has been successfully employed in a vaccine context and has a good safety record. This gives impetus to the rationale for using adenoviruses as vectors for human use. Michel Perricaudet (Paris) outlined the general strategies behind the current generation of vectors, which again depend heavily on methodology that has been in laboratory use for a number of years. The basic design renders the vector replication incompetent by deleting the essential E1 region. Expression of this region is a prerequisite for expression of the rest of the viral genome, and thus an E1-deficient recombinant should express only the therapeutic gene of interest when delivered to the target cell. The recombinants themselves are constructed and propagated in cells that stably express the E1 region, thus allowing virus stocks to be prepared. In addition to the E1 deletion, extra space for the insertion of foreign genes can be generated by deleting the E3 region, which has been demonstrated to be non-essential for growth in cell culture. Alan Smith (Framingham) outlined an alternative strategy in which, along with E1, portions of the E4 region are deleted, leaving only the E4 open reading frame 6 intact. These vectors grow well, although the full potential of vectors containing simultaneous deletions in E1, E3 and E4 has yet to be explored.

Ken Berns (New York) described adenovirus-associated virus (AAV). This small human parvovirus can replicate only in the presence of adenovirus or herpes simplex to provide replicative helper functions. It has found some utility as a vector and has a number of attractive features, including lack of disease associations or superinfection immunity, ability to infect different types of cells and the ability to infect non-dividing cells. It has a foreign DNA capacity of about 5 kb. In the absence of helper virus, AAV and AAV vectors integrate into the host genome at a limited number of sites, reducing the probability of random, deleterious insertional mutagenesis. Thus, like retroviruses, they lead to persistence of the introduced gene in an integrated state. They can occasionally exhibit high transduction frequencies of the order of 50–80%.

Diseases that involve cells of the nervous system (e.g. neurodegenerative diseases) are currently receiving increased attention. Herpes simplex virus (HSV) is an attractive vector for neurological targeting since it naturally establishes latency in neurons of the peripheral nervous system with the concomitant expression of latency-associated transcripts and loss of lytic functions. Joe Glorioso (Pittsburgh) recounted his team's attempts to exploit these features as a means of developing brain delivery systems. Two major hurdles have initially to be overcome: (1) viral cytotoxicity and (2) persistence of foreign gene expression. The extensive molecular genetic knowledge of HSV has allowed significant progress to be made in addressing the first problem by the use of virus deletion mutants and appropriate complementing cell lines coupled with further deletions in some of the large number of genes known to be non-essential for growth *in vitro*. Several approaches to overcome the difficulty of obtaining persistent gene expression have been tried. One promising solution appears to be the use of a recombinant yeast GAL4–VP16 fusion gene that is capable of sustained activation by transactivation of its own promoter.

However, viruses are not the only means of transferring genes to target tissues, although they can be used as accessory agents in some other forms of delivery. One such instance is the receptor-mediated gene delivery system described by Matt Cotten (Vienna). This consists of the DNA to be transfected, condensed with a polycation (e.g. polylysine), inclusion of a cell receptor-recognising component (e.g. transferrin) and streptavidin. Chemically inactivated, biotinylated adenovirus particles are bound to the complex via the streptavidin–biotin interaction and provide an endosome disruption activity required for exit of the complex from endosomes. The transfection process appears to be efficient and, since the DNA is not subject to the size constraints imposed by a viral vector system, can be employed to transfer large segments of genetic information. In addition variation of the cell-binding agent offers the prospect of a degree of cell targeting.

A second non-viral method of gene delivery, liposomes, was discussed by Bob Williamson (London). The major advantages of this approach are the apparent lack of foreseeable hazard as compared with viral delivery systems and, again, the absence of size constraints on the sequences which can be delivered. On the other hand, relatively poor transfection efficiency compared with virus systems means that the amounts of DNA which must be incorporated are large. Secondly since the transfected DNA is not integrated into the genome, expression is likely to be short lived. Also, the potential toxicity of liposomes has not yet been fully evaluated. Animal model systems indicate that various formulations of liposomes are effective, when used either topically or after nebulisation, at introducing gene sequences which can be expressed into cells lining the lung airways. Recently this has been extended to a liposome-mediated gene transfer trial for cystic fibrosis in humans.

A second human trial aimed at correcting the cystic fibrosis (CF) defect was described by Alan Smith. *In vitro* experiments demonstrated that relatively low multiplicities of infection of CF cells in culture by a recombinant adenovirus would correct both the fluid transport and electro-

*Meeting held 31 October to 3 November.

physiological defects of the cells. A trial was devised whereby a small area of the nasal epithelium was infected with the recombinant. Expression of the gene and correction of electrophysical defects in the treated cells was demonstrated.

Recombinant adenovirus has also been used in a mouse model of muscular dystrophy. Perricaudet described a recombinant containing a 6.3 kb Becker-like dystrophin cDNA driven by the Rous sarcoma virus (RSV) promoter. A single intramuscular injection into 5- to 9-day-old dystrophin-deficient mdx mice resulted in expression of the dystrophin gene within the muscle cells for at least 6 months. In addition, the treated muscles were protected against degeneration.

An alternative *ex vivo* approach for muscular dystrophy, that of genetically correcting and retransplanting myogenic stem cells, was discussed by Terry Partridge (London). The attraction of this approach is that progressive repopulation of dystrophic muscle by the transduced myoblasts might eventually lead to genetic correction of whole muscle groups, circumventing one of the current problems with *in vivo* gene therapy of muscular dystrophy, namely that the extent of genetic correction seen in a muscle is generally limited to a small area around the site of administration of the therapeutic agent. Using a dystrophic mouse model he presented some evidence for such a primitive muscle progenitor cell capable of *in vivo* reconstitution and phenotype correction, and this approach may prove of some benefit in treating the major skeletal muscle groups.

To facilitate long-lived correction of a genetic defect it will be advantageous to target stem cells. Chris Potten (Manchester) defined stem cells as self-renewing, proliferative cells with a large division potential capable of producing many progeny which may differentiate in a variety of ways. *In vitro* manipulation of stem (or at least very primitive progenitor) cell populations has proved possible for a number of systems, including haemopoietic, gut and muscle tissues. Ron McKay (NIH) described immortalised lines derived from nestin (a neural 'stem cell'-specific gene) positive cells which may be propagated *in vitro*, and when implanted into the developing hippocampus differentiate *in vivo* both morphologically and functionally and are synaptically integrated with the host brain. Cells such as this may prove useful targets for genetic therapy of brain disorders. However, there are difficulties in working with unimmortalised stem cells. These include their identification, targeting, small numbers, accessibility and slow cycle times (or even quiescence).

Wolfram Ostertag (Hamburg) discussed two areas which restrict the efficiency of retroviral-mediated gene transfer into haemopoietic stem cells. The first concerns the need to maintain the number and quality of progenitor cells in explanted material. Although cocktails of soluble growth factors may expand target cell populations, a net differentiative effect is often seen. Co-culture of target cells with stroma in the absence of added factors was found to be the best method of maintaining the quality of stem cells. The use of long-term bone marrow cultures provides just such conditions and may facilitate manipulation of haemopoietic stem cells (Leslie Fairbairn, Manchester).

A further issue is development of retroviral vectors suitable both for high-titre infection of stem cells and maintenance of gene expression in their progeny. Ostertag demonstrated the value of forced retroviral passage in the selection of beneficial mutations, such as those which overcome the down-regulation of long terminal repeat (LTR) function found in primitive cells. The application of this technique to recombinant retorviruses promises further increases in the efficiency and repertoire of retroviral vectors.

For many applications it is desirable to direct the expression of introduced genes to a specific subset of the recipient population. In this respect, studies of transcriptional control reported by Dan Tenen (Boston) and Frank Grosveld (Rotterdam) provided indications of the multiple levels at which transcriptional selectivity may be influenced. Tenen described the involvement of the PU.1 and SP1 transcription factors in directing the macrophage-specific expression of the CD11b and macrophage colony-stimulating factor (M-CSF) receptor genes. It is hoped that regulatory elements of this type will be invaluable in modulating the expression pattern of linked therapeutic genes. However, we still know very little about the establishment of gene transcription patterns during the developmental processes which will be relevant to many gene therapeutic approaches. The presentation of Grosveld described multiple levels at which the transcription patterns of at least some endogenous genes are determined. Studies on the developmental regulation of the beta-globin gene locus have demonstrated a requirement for regulatory elements a large distance from their target genes. These 'locus control regions' (LCRs) consist of dispersed groups of transcription factor binding sites, and are absolutely required to establish high-level, specific transcription in developing cells. The activity of regulatory regions located close to each gene of the beta-globin cluster is dependent upon interaction with LCR elements in a competitive manner. There is much still to learn about the involvement of long-range regulatory interactions and higher order chromatin structure in the establishment and maintenance of tissue-specific gene expression. Furthermore, it seems likely that the combination of elements required to achieve tissue-specific and prolonged expression of some genes may lead to a need to use larger therapeutic DNA sequences than can be accommodated in a retrovirus.

An obvious role for gene therapy is in the treatment of inherited genetic disorders, many of which are caused by single-gene defects. Indeed, the first therapeutic use of gene therapy was for the rare autosomal recessive disorder, severe combined immune deficiency (SCID), a consequence of defects in the gene encoding the enzyme adenosine deaminase (ADA) and which results in lymphopenia and deficits in both humoral and cellular immunity. Jay Ramsay (NIH) described these first treatments, which used retroviral gene transfer of a normal human ADA cDNA into peripheral T cells. The two patients in the trial have elevated ADA levels in peripheral blood lymphocytes which have been sustained for up to 12 months following infusion. Functionally, their immune systems show significant reconstitution with decreased frequencies and severities of infectious diseases and improved responses to immune challenges. These encouraging data have led to the development of strategies for haemopoietic stem cell gene therapy of this disease to achieve long-term improvement of immune function without the need for continued reinfusion.

Haemopoietic stem cell gene therapy is also the method of choice for other inherited disorders of the blood system. Mike Antoniou (London) addressed the problems associated with a gene therapy approach to the haemoglobinopathies ($\alpha$- and $\beta$-thalassemia, sickle cell anaemia). Although, given the complexities associated with controlled expression of globin genes, a gene therapeutic answer to these diseases may still be a long way off, the widespread nature of the haemoglobinopathies (compared with the relative rarity of, for example, ADA-SCID) makes a powerful case for substantial technological investment in their treatment.

The haemopoietic stem cell lends itself as a target for gene therapy of diseases whose effects are not limited to cells of the haemopoietic system. Thus, where a defective gene encodes a secreted protein, expression and subsequent secretion of a product by haemopoietic cells may result in alleviation of disease. Examples of this are the lysosomal storage disorders, in which lack of one enzyme leads to the accumulation of glycosaminoglycans in the lysosomes of cells and to a range of clinical effects. Because enzyme secreted by one cell can be sequestered by non-expressing cells and targeted to their lysosomes, the cells of the haemopoietic system can act as producers of enzyme for uptake by other tissues. Furthermore, the ability of tissue macrophages to enter the brain means that this approach might circumvent the blood–brain barrier and hence effect transfer of enzyme to neurons and other cells of the central nervous system. Allogeneic bone marrow transplantation has already been used in this context for Hurler's syndrome, and this approach is able to alleviate many aspects of the disease phenotype, including neurological effects. Fairbairn presented work showing that long-term (30 weeks) expression and secretion of the enzyme α-L-iduronidase and apparent correction of disease phenotype *in vitro* is possible after retroviral gene transfer to bone marrow cells from patients with Hurler's syndrome.

When there is no significant neurological involvement in a disorder, and thus no need for delivery of product across the blood–brain barrier, other tissues also lend themselves as targets for transfer of genes encoding secreted proteins. Oliver Danos (Paris) described such an approach for another lysosomal storage disorder, mucopolysaccharidosis (MPS) VII (Sly syndrome). Primary fibroblasts from MPS VII mice were transduced with a retrovirus expressing human β-glucuronidase, embedded in a collagen lattice and reimplanted. Danos and colleagues saw vascularisation of the neo-organ, accumulation of enzyme in some tissues and correction of the storage lesions in those tissues. Scaled-up experiments using a dog model of the disease suggest that this approach is feasible for larger animals. In a similar approach, Inder Verma (San Diego) and colleagues have used gene transfer into myoblasts in developing a gene therapy approach to the clotting disorders. They found that myoblasts can be readily infected *in vitro* with retroviruses, and that following fusion of the myoblasts *in vivo* to form myotubes expression of the foreign protein (in this case human factor IX) was maintained for up to 15 months in recipient mice without any apparent adverse effects, raising genuine prospects for gene therapy of these diseases. Both Verma and Danos noted that in order to maintain expression in post-mitotic cells (fibroblasts and myotubes) it was necessary to use promoters other than the retroviral LTR. Thus the use of a phosphoglycerate kinase promoter for fibroblasts and a muscle creatine kinase promoter for myoblast gene therapy was necessary for sustained expression. This restriction of expression, which may reflect tissue-specific expression of transcription factors, will be of importance to other strategies aimed at using such cells for exocrine gene therapy, particularly where it may be desirable (as in the case of insulin gene therapy for diabetes) to obtain modulation of expression in response to physiological changes.

Diseases which are due to acquired genetic defects (notably cancers) are also tempting targets for genetic therapy. There are treatment regimens available for most tumours, which give either a full or partial cure of disease in a fraction of patients that may be a large proportion of those presenting with disease or a depressingly low one. As Howie Scarffe (Manchester) pointed out, the wealth of knowledge acquired to date about the nature of cancer has not yet led to a marked improvement in clinical results, and most efficacious chemotherapeutic treatments (particularly those of childhood cancer, lymphoma and leukaemia) have been arrived at in an empirical and pragmatic way. Improvements in support care of patients, refined methods of surgery, radiotherapy and clinical use of haemopoietic growth factors or bone marrow/peripheral blood progenitor cells transplants have led to decreased patient morbidity and mortality. Nevertheless the rate of cure of some of the most common adult cancers (e.g. breast and colon) has remained largely unchanged. He ended with a call for novel approaches to cancer treatment.

David Lane (Dundee) discussed the possibility of using one of the genetic defects of a tumour as a target for genetic therapy of disease. A common feature of many different tumours is loss of function of a specific gene product, and a number of these commonly lost genes are tumour suppressors, of which the retinoblastoma and the p53 genes are the best-known examples. Restoration of wild-type p53 function to tumour cells can lead to dramatic growth-inhibitory or lethal effects. A further feature of normal p53 is that it is markedly more stable in tumour cells than in normal cells. Thus its expression is well tolerated by normal cells, whereas it accumulates in tumour cells, promoting a profound growth arrest or cell death via apoptosis. Thus it might prove possible to use p53 gene transfer in a therapeutic situation to obtain arrest or decline of a tumour while the effects upon fortuitously transfected normal cells could prove minimal.

One of the critical steps in the development of a tumour may be the ability of the abnormal cells to escape immune surveillance. As a result, tumour cells are often poor immunogens and attempts are being made to modulate their immunogenicity by inducing them to express and secrete cytokines which can stimulate cells of the immune system. Mary Collins described comparisons of the ability of interleukin 2 (IL-2), IL-4 and γ-interferon gene expression in tumour cells to induce rejection of admixed unmodified tumour cells. In their system, γ-interferon offered the best hope of tumour rejection compared with IL-2, which has proved efficacious in other animal model systems. However, the acid test, that of treatment of tumour *in situ*, is yet to be done. In a rat model of metastatic disease, Collins showed the efficacy of IL-2-secreting tumour cells in preventing the development of secondary tumours following excision of the primary lesion. This approach may hold real hope for cytokine gene therapy in an adjuvant role.

A major problem with chemotherapeutic approaches to cancer treatment is the dose-limiting toxicity of most anti-tumour agents, and approaches to reduce the cytotoxic effects in normal tissues while maintaining the sensitivity of tumour cells to cytotoxic agents would be useful. A number of genes are available which confer resistance to cytotoxic agents. Massimo Gianni (Milan) and Joe Rafferty (Manchester) described similar approaches using different resistance genes. Gianni discussed the efficacy of the human aldehyde dehydrogenase (*ALDH-1*) gene to confer resistance to the alkylating agent cyclophosphamide. K562 cells induced to express *ALDH-1* are 3–5 times more resistant to cyclophosphamide than controls. The product of the $O^6$-alkyl-DNA alkyltransferase (ATase) gene confers resistance to chemical alkylating agents, a large family of cytotoxics, many of which are used clinically. Rafferty showed that expression and persistence of a transferred ATase gene in primary murine haemopoietic cells could be achieved via retroviral gene transfer. ATase expression conferred resistance to the cytotoxicity of *N*-methyl-*N*-nitrosourea (MNU), suggesting such an approach could be used to alleviate secondary toxicity following treatment with alkylating agents such as bis-chloroethylnitrosourea (BCNU). Many tumours also express ATase, accounting for their relative resistance to alkylating agent-induced death. Inhibitors of ATase, such as $O^6$-benzylguanine (BzG), can reduce this resistance, but also affect normal tissues. Retroviral gene transfer of the *E. coli* ATase (*ada*), which is more resistant to BzG than the endogenous ATase, into sensitive normal tissues raises the possibility of specifically increasing the resistance of bone marrow (or lung, etc.) to a chemotherapeutic regimen while increasing the sensitivity of tumour cells. When designing gene therapy approaches to haemopoietic toxicity, consideration should be given to the target cell population which needs to be protected. Perhaps stem cells are relatively resistant to alkylating agents owing to their proliferative quiescence and the most at-risk populations of haemopoietic cells are the progenitor cells. If so, peripheral blood progenitor cells, which can be mobilised by chemotherapy and cytokines, and which are actively cycling and therefore targets for recombinant retroviruses, may be the population to which this approach may be most appropriate. Gianni presented evidence that under appropriate circumstances nearly 100% of colony-forming cells from the peripheral blood of a chemotherapy/cytokine-treated patient may be infected with a retroviral vector.

An alternative to protecting sensitive normal tissues from the cytotoxic effects of anti-cancer drugs is to avoid their exposure to such agents. One approach to this would be to modify tumour cells such that they specifically activate an otherwise harmless prodrug to generate the cytotoxic derivative only inside the tumour cell. Karol Sikora (London) described the use of a c-*erb*B-2 promoter to drive expression of a cytosine deaminase gene in transduced tumour cells. Transcription of c-*erb*B-2 is much greater in some tumours and is associated with the presence of a transcription factor which binds to a response element within the c-*erb*B-2 promoter. Thus it may prove possible to achieve high levels of cytosine deaminase in some tumour cells compared with normal tissues. Since cytosine deaminase converts 5-fluorocytosine (5-FC) to the cytotoxic agent 5-fluorouracil, it follows that such cells would be selectively killed after administration of 5-FC to a patient.

In a similar approach, Zvi Ram and colleagues (NIH) have used retroviruses carrying the herpes simplex virus thymidine kinase (HStk) gene to express this gene in tumour cells. In animals models of brain tumours, they were able to obtain transfer and expression of HStk in the cycling neoplastic cell population after direct injection of retroviral producers into the tumours. The quiescent normal cells of the brain appeared largely unaffected, and following treatment with the prodrug pre-established tumours were seen to regress. Subsequently a clinical trial treating eight patients with unresponsive, progressive malignant brain tumours has been initiated. In five of the eight patients treated some evidence of an anti-tumour response (decrease in tumour size or changes in tumour consistency) was seen. Although this trial was not intended as a full therapeutic treatment but was designed to evaluate toxicity and the potential for anti-tumour activity *in vivo* in humans, the data thus far look extremely promising with anti-tumour effects being achieved without any treatment-associated toxicity being observed. Based on data from animal models, it is unlikely that all the tumour cells which were killed were transduced with the HStk retrovirus. This phenomenon, known as the bystander effect, in which induction of death in some cells leads to death of surrounding ones, is not well understood. However, it is likely that a number of effects, including transfer of activated drug via gap junctions or by intercellular diffusion, destruction of tumour microcirculation and activation of an immune response, may contribute to the cell death seen.

The meeting ended with a session devoted to regulatory, ethical and safety considerations. Sir Cecil Clothier (Chairman of the first government-appointed committee to examine the implications of gene therapy) pointed out that all attempts to restrain or confine the progress of science are contrary to man's nature and therefore will fail. However, there is always the temptation to abuse scientific developments, the motive usually being profit. The more blatant of such abuses drive society to legal regulation. Clothier asserts that generally new technology is available only to a small number of individuals who are of high integrity but that, as time goes by, technology advances and becomes more widely available, at which time the abusers move in. Therefore regulation can and should be voluntary at first, but once the scope for abuse is determined legal controls should be applied.

John Harris (Manchester) considered ethical issues, in particular the distinction between somatic and germ-line therapy. The general perception had been that these forms of therapy are fundamentally different from an ethical point of view and should be regulated differently. However Clothier clarified his committee's thesis that rather than being an ethical issue, the current distinction between the two was on the grounds of current lack of availability of knowledge of the consequences of gene therapy and that as more became known we would be better able to contemplate germ-line therapy.

Tony Meager (Potter's Bar) concluded the presentations with a sobering enumeration of the extensive requirements of safety regulatory authorities' demands for vector and product characterisation, stringent testing for a large number of undesirable agents, criteria for containment and operating standards of production facilities and the need for comprehensive documentation of reagents and procedures.